# Effects Comparison between Endoscopic Papillary Large Balloon Dilatation and Endoscopic Sphincterotomy for Common Bile Duct Stone Removal

**DOI:** 10.1155/2015/839346

**Published:** 2015-08-17

**Authors:** Yandong Guo, Chen Li, Shan Lei, Fachao Zhi

**Affiliations:** Department of Gastroenterology, Guangdong Provincial Key Laboratory of Gastroenterology, Nanfang Hospital, Southern Medical University, Guangzhou 510515, China

## Abstract

Endoscopic sphincterotomy (EST) is a treatment of choice for stone extraction and is now most frequently used. The study was to compare the efficacy of endoscopic papillary large balloon dilatation (EPLBD) and endoscopic sphincterotomy (EST) for common bile duct stone removal. Trials comparing the effects between EPLBD and EST treatment were searched according to the study protocol. Overall stone removal rate, complete removal rate in 1st session, treatment duration, mechanical lithotripsy using rate, and overall complication rate were compared using risk ratio (RR) and mean difference (MD) and their 95% confidence interval (CI) via RevMan 5.2 software. For overall stone removal rate, two therapies showed similar effect, but EPLBD showed better overall stone removal rate for stone >10 mm in diameter. For complete stone removal rate in 1st session, no difference was found, even for those with stone >10 mm in diameter; EPLBD showed longer treatment duration, higher mechanical lithotripsy using rate obvious overall complications rate, and more serious bleeding, whereas there were no significant differences for perforation, hyperamylasemia, pancreatitis, and cholecystitis/cholangitis. EPLBD showed better efficacy in certain conditions compared to EST, however with shortcomings, such as more duration, higher mechanical lithotripsy using rate, more serious overall complications rate, and bleeding.

## 1. Introduction

Common bile duct stones are present in about 4–10 percent of patients who have undertaken cholecystectomy [1]. Reported incidence of common bile duct stone varies from 5% to 11% at the time of cholecystectomy [2, 3]. The vast majority of common bile duct stone mainly originates from gallbladder [4]. Its signs and symptoms are variable, ranging from being completely asymptomatic to complications, such as biliary colic, cholangitis, jaundice, or pancreatitis [5].

Endoscopic sphincterotomy (EST), first described in 1974, is a treatment of choice for stone extraction and is now most frequently used [6]. Although EST is relatively safe, it is reported to induce high level of overall complication rates, as well as short-term risks, including bleeding, perforation, and pancreatitis. Endoscopic papillary large balloon dilatation (EPLBD) had been introduced as an alternation for EST to manage the common bile duct stones, for its lower frequency in perforation and hemorrhage [7]. However, in another study comparing these two treatments, EST showed lower pancreatitis occurrence than EPLBD and 2 patients died of pancreatitis because of the EPLBD extraction [8]. It remained as a controversial issue whether EPLBD has a better effect than EST.

In this study, we conducted a meta-analysis to compare the data collected in trials published between 2004 and 2013. Overall stone removal rate, complete stone removal rate in 1st session, treatment duration, mechanical lithotripsy using rate, and overall/each complications rate were compared between the two therapies.

## 2. Methods

### 2.1. Information Sources and Searches

A search of the literature was conducted for studies that reported the EPLBD versus EST for common bile duct stone removal. The PubMed, Web of Science, Medline, Science Citation Index, EMBASE, China National Knowledge Infrastructure, Wanfang Database, and China Biomedical Database were searched to identify double blinded research (D, S, R), single center trials (S), and random clinical trials (RCTs published) in the field of common bile duct stone removal between 2004 and 2013. The keywords used in literature searches included the following: bile duct stones, endoscopic sphincterotomy, and endoscopic papillary balloon dilatation.

### 2.2. Eligibility Criteria and Outcome Measure

The inclusion criteria were the following: (i) the included studies were designed to compare the therapeutic effects of endoscopic sphincterotomy and endoscopic papillary large balloon dilatation or the combination of the two therapeutics and (ii) the publications could be written in any language. Reports of duplicated studies were excluded by examining the author list, parent institution, sample size, and results.

The primary outcome was overall stone removal rate, overall/complete stone removal rate in 1st session, treatment duration, mechanical lithotripsy using rate, and overall/each complications rate.

### 2.3. Assessment of Study Quality

Two authors (Shan Lei and Chen Li) independently assessed the quality of the included studies according to the descriptions provided by the authors of the included trials. The methodological quality of the trials was assessed based on adequate sequence generation, allocation concealment, blinding, management of incomplete outcome data, and early stopping for benefit [18].

### 2.4. Study Selection and Data Collection

Two authors (Shan Lei and Chen Li) independently screened titles and abstracts for potential eligibility and the full texts for final eligibility. We extracted the data from the included trials independently for quantitative analyses, and any disagreement was subsequently resolved by discussion. The quantitative data included the country, stone diameters, interventions, and dilated balloon catheter.

### 2.5. Synthesis of Results

In this meta-analysis, we used a random effect model because of the anticipated variability among trials with regard to patient populations [19, 20]. The measure of association used in this meta-analysis was the risk ratio (RR) or mean difference (MD) with a 95% confidence interval (CI). The summary RR with the 95% CI was calculated by the RevMan 5.2 software using the random or fixed effect model. A statistically significant result was assumed when the 95% CI did not include one.

Heterogeneity was explored using a Chi-square test; *P* < 0.05 represents that there is heterogeneity of effect size. In addition, the quantity of heterogeneity was measured using the *I* ^2^ statistic, which calculates the percentage of total variation across studies caused by heterogeneity rather than sampling errors. *I* ^2^ = 25% was considered as low heterogeneity and 50% as moderate, while 75% was considered as high.

## 3. Results

### 3.1. Literature Search and Population

Fifteen trials were finally identified in the English literature. Two trials were removed after blind review because they did not compare the result of EST and EPLBD, 2 reviews were also excluded, and the data of 1 literature was also excluded for the information being incomplete. Characteristics of the final 10 prospective studies [6, 9–17] are shown in Table 1. Patient numbers with EST and EPLBD treatment were 763 and 769 (Table 1).

### 3.2. Comparison of the Overall Stone Removal Rate of EPLBD and EST Treatments

**Figure 1:**
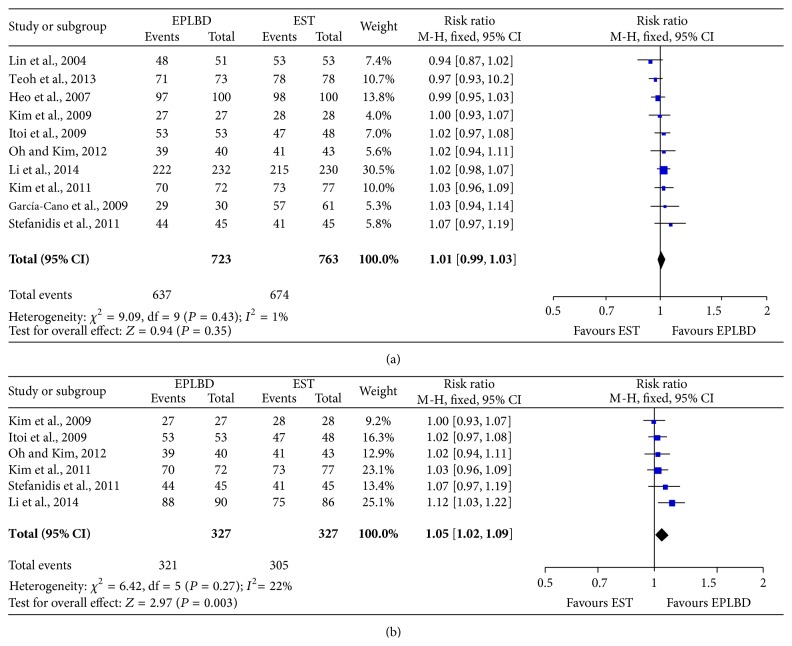
Rate ratio for overall stone removal rate of EPLBD and EST treatment (a) and overall stone removal rate for stone >10 mm in diameter (b).

**Figure 2:**
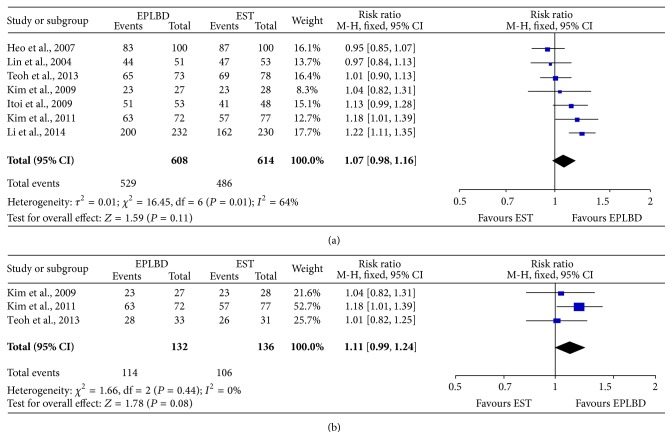
Rate ratio for complete stone removal rate in 1st session of EPLBD and EST treatment (a) and complete stone removal rate in 1st session for stone >10 mm in diameter (b).

The rate ratios for overall stone removal rate for patients taking EPLBD therapies were similar to that of those taking EST therapies (RR: 1.01, 95% CI: 0.99–1.03; *P* = 0.35) (Figure 1(a)), but, for the patient with stone >10 mm in diameter, the rate ratios for overall stone removal rate of EPLBD were higher than those of EST (RR: 1.05, 95% CI: 1.02–1.09, *P* < 0.05) (Figure 1(b)). The result showed that EPLBD had better efficacy than EST method for stones larger than 10 mm in terms of overall removal rate. The rate ratio for complete stone removal rate in 1st session of EPLBD was similar to that of EST (RR: 1.07, 95% CI: 0.98–1.16, *P* = 0.11) (Figure 2(a)), and the rate ratio for complete stone >10 mm in diameter removal rate in 1st session for EPLBD and EST was also similar (RR: 1.11, 95% CI: 0.99–1.24, *P* = 0.08) (Figure 2(b)). Thus, there was no significant difference between the two kinds of treatment in terms of complete stone removal rate in 1st session. In this meta-analysis for complete stone >10 mm in diameter removal rate in 1st session, there was apparent heterogeneity (*P* = 0.01, *I* ^2^ = 64%), so random effect model was used. What is more, for the other outcomes, fixed effect model was used for there was no apparent heterogeneity.

### 3.3. Comparison for Treatment Duration and Mechanical Lithotripsy Using Rate for EPLBD and EST

**Figure 3:**
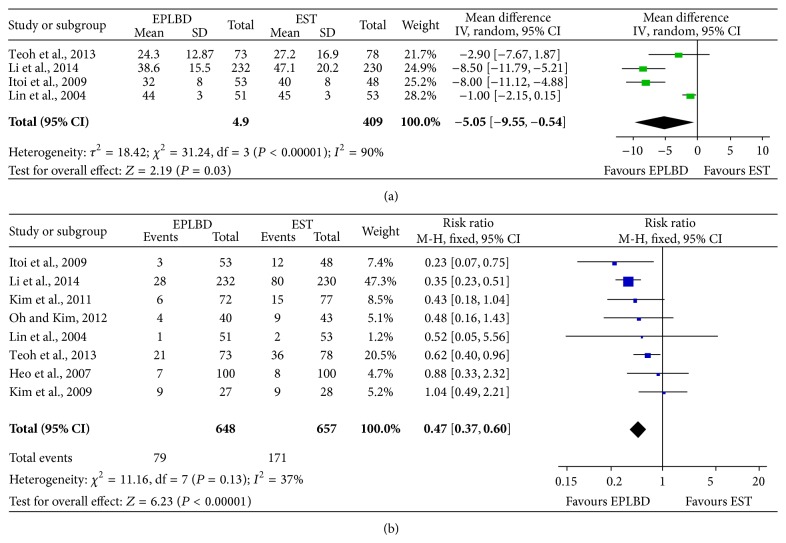
Comparison of treatment duration (a) and mechanical lithotripsy (b) using rate of EPLBD and EST treatment.

There was significant heterogeneity in the assessment of treatment duration (*P* < 0.00001, *I* ^2^ = 90%), so random effect model was used. It turned out that the two kinds of treatment were significantly different (MD = −5.05, 95% CI: −9.55~−0.54, *P* = 0.03; Figure 3(a)), and EPLBD showed longer treatment duration. Fixed effect model was used for mechanical lithotripsy assessment for no heterogeneity (*P* = 0.13, *I* ^2^ = 37%), and the RR value was 0.47 with 95% CI being between 0.37 and 0.60 (*P* < 0.00001; Figure 3(b)). EPLBD treatment had higher mechanical lithotripsy using rate compared with EST treatment.

### 3.4. Comparison for Complications Rate for EPLBD and EST

**Figure 4:**
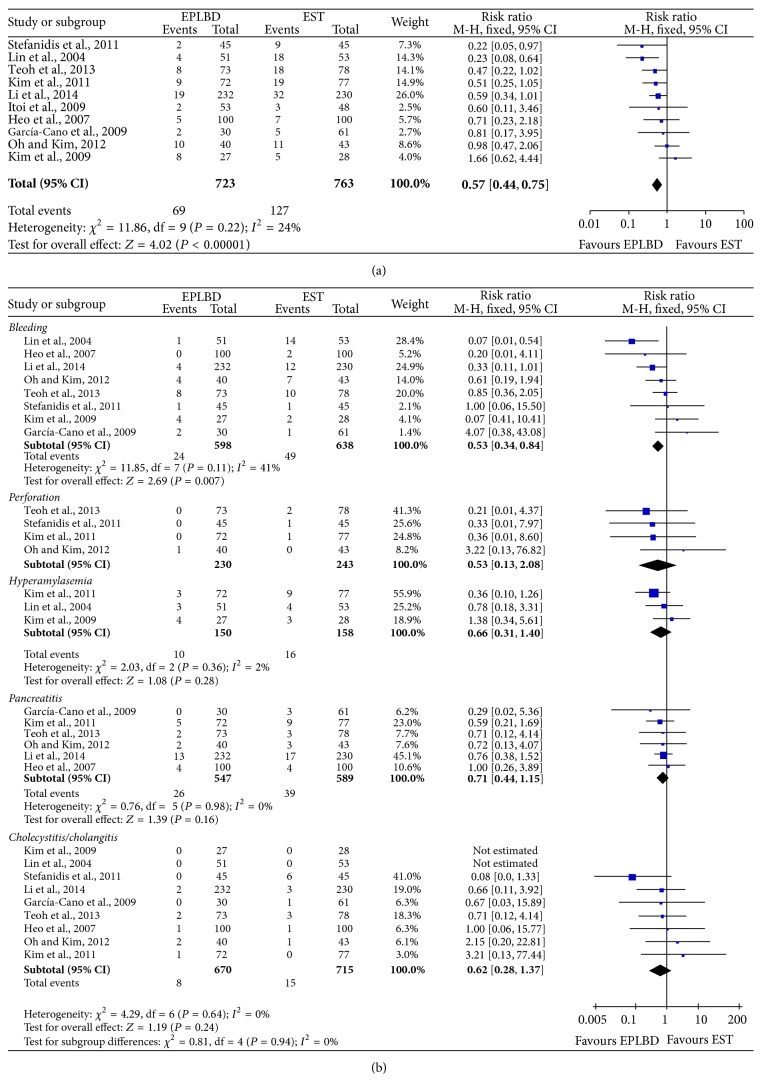
Comparison of overall complications rate (a) and each complications rate (b) of EPLBD and EST treatment.

No apparent heterogeneities were detected between the studies in terms of overall complications rate (*P* = 0.22, *I* ^2^ = 24%) and each complications rate (bleeding: *P* = 0.11, *I* ^2^ = 41%; perforation: *P* = 0.63, *I* ^2^ = 0%; hyperamylasemia: *P* = 0.22, *I* ^2^ = 24%; pancreatitis: *P* = 0.98, *I* ^2^ = 0%; cholecystitis/cholangitis: *P* = 0.64, *I* ^2^ = 0%); then fixed effect model was used for the meta-analyses. EPLBD showed obvious overall complications rate (RR = 0.57, 95% CI = 0.44~0.75, *P* < 0.0001; Figure 4(a)) and more serious bleeding (RR = 0.53, 95% CI = 0.34~0.84, *P* = 0.007; Figure 4(b)) than EST, while there were no significant differences between these two treatments for perforation (RR = 0.36, 95% CI = 0.13~2.08, *P* = 0.36), hyperamylasemia (RR = 0.66, 95% CI = 0.31~1.40, *P* = 0.28), pancreatitis (RR = 0.71, 95% CI = 0.44~1.15, *P* = 0.16), and cholecystitis/cholangitis (RR = 0.62, 95% CI = 0.28~1.37, *P* = 0.24) (Figure 4(b)).

## 4. Discussion

EST used to be the most commonly used treatment for the removal of bile duct stones. Nevertheless, it is commonly reported that EST could induce substantial procedure associated risks, such as increased incidence of ascending cholangitis [6]. Endoscopic papillary balloon dilatation uses a small balloon catheter, while EPLBD uses a larger balloon (>12 mm) after the mid-incision EST to remove large common bile duct stones. EPLBD would theoretically combine the advantages of balloon dilation and sphincterotomy by increasing stone extraction efficacy while minimizing complications of them. We performed this meta-analysis to compare the effect of EST and EPLBD for common bile duct stones. Even no significant difference existed in the overall stone removal rate, but, for patients with stones >10 mm in diameter, EPLBD could give better overall stone removal rate; however, this treatment needs more duration and will induce higher mechanical lithotripsy using rate with more serious overall complications rate and bleeding, compared with EST. The result was consistent with the study of Fujita et al. [21]. Nevertheless, many studies have recently proposed that EPLBD will not cause complications if performed under strictly established guidelines [22]. Thus, it is of great importance to follow the guidelines during the treatment of EPLBD.

We reviewed previous meta-analysis and found that patients received EPLBD therapy which was associated with shorter length of hospitalization, whereas there was no significance for clearance rates and morbidity/mortality [4]. However, these conclusions may be unreliable, because only 2 included trials reported these outcomes. The current study is based on 10 prospective studies, which are potentially more robust than previous meta-analysis.

There are few limitations of our current study: (1) not all the included literatures studied all the assessment parameters, such as complete stone removal rate in 1st session and treatment duration; (2) the trials adapted different design, including D, S, R, and R; (3) we did not conduct sensitive analysis to test the reliability of the results.

In conclusion, the EPLBD treatment needs more treatment duration and mechanical lithotripsy using rate compared to EST, but, for patients with stones >10 mm in diameter, EPLBD could give better overall stone removal rate. What is more, EPLBD would produce obvious overall complications rate and bleeding, compared with EST.

## Figures and Tables

**Table 1:** Characteristics of the included 10 prospective studies.

Study	Design^*∗*^	Country	Bile duct stones	Intervention (*n*)	Dilated balloon catheter
Lin et al., 2004 [9]	D, S, R	China	Multiple, mean = 8 mm	EPLBD (51) or EST (54)	10–12 mm
Heo et al., 2007 [6]	D, S, R	Korea	Multiple, mean = 16.0–15.0	EST plus EPLBD (100) or EST (100)	12–20 mm
Itoi et al., 2009 [10]	S	Japan	Large	Small EST plus EPLBD (53) or EST (48)	10–20 mm
García-Cano et al., 2009 [11]	D, S	*Spain *	Multiple, mean = 3 mm	EST plus EPLBD (31) or EST (60)	10–20 mm
Kim et al., 2009 [12]	D, S, R	South Korea	Large, ≥15 mm	Small EST plus EPLBD (27) or EST (28)	15, 16.5, or 18 mm
Kim et al., 2011 [13]	S	Korea	Large, ≥10 mm	Small EST plus EPLBD (72) or EST (77)	12–20 mm
Stefanidis et al., 2011 [14]	D, S, R	Greece	Large, >12 mm	Full EST plus EPLBD (45) or EST plus ML (45)	10–20 mm
Oh and Kim, 2012 [15]	S, R	Korea	Large, >10 mm	EPLBD (40) or EST (43)	10–20 mm
Teoh et al., 2013 [16]	D, S, R	China	Large, >13 mm	Limit EST plus EPLBD (73) or EST (78)	15 mm
Li et al., 2014 [17]	S, D	China	Multiple, mean = 12.7–13.2	Small EST plus EPBD (232) or EST (230)	15, 16.5, or 18 mm

^*∗*^D: double blinded; S: single center; R: randomization.
